# Evaluation of machine learning methods for the retrospective detection of ovarian cancer recurrences from chemotherapy data

**DOI:** 10.1016/j.esmorw.2024.100038

**Published:** 2024-05-01

**Authors:** A.D. Coles, C.D. McInerney, K. Zucker, S. Cheeseman, O.A. Johnson, G. Hall

**Affiliations:** 1 School of Computing, University of Leeds, Leeds, UK; 2 School of Medicine & Population Health, University of Sheffield, Sheffield, UK; 3 School of Medicine, University of Leeds, Leeds, UK; 4 Leeds Cancer Center, Leeds Teaching Hospitals NHS Trust, Leeds, UK

**Keywords:** cancer recurrence, chemotherapy, electronic health record, machine learning, artificial intelligence

## Abstract

**Background:**

Cancer recurrences are poorly recorded within electronic health records around the world. This hinders research into the efficacy of cancer treatments. Currently, the retrospective identification of recurrence/progression diagnosis dates is achieved by staff who manually review patients’ health records. This is expensive, time-consuming, and inefficient. Machine Learning models may expedite the review of health records and facilitate the assessment of alternative cancer therapies.

**Materials and methods:**

This paper evaluates the use of four machine learning models (random forests, conditional inference trees, decision trees, and logistic regression) in identifying proxy dates of epithelial ovarian cancer recurrence/progression from chemotherapy data, in 531 patients at Leeds Teaching Hospital Trust.

**Results:**

The random forest achieved the highest F1 score of 0.941 (95% confidence interval 0.916-0.968) when identifying recurrence events. Both the random forest and decision tree models’ classifications closely conform to chart-reviewed time to next treatment, serving as a surrogate for recurrence-free survival. Additionally, all models reached an F1 score >0.940 when identifying patients whose cancer recurred/progressed.

**Conclusions:**

Our models proficiently identify both proxy dates for recurrence/progression diagnoses and patients whose cancer recurred/progressed. Considering the similar performance of the random forest and decision tree, model preference should be determined by the interpretability required to assist chart review and the ease of implementation into existing architecture.

## Highlights

• Our models accurately identify both initial and subsequent cancer recurrences.
• Models can use chemotherapy alone to identify recurrence dates.
• Survival informed from model-identified dates matches chart review survival.

## Introduction

The recurrence of a patient’s cancer is a clinically significant event, enabling the measurement of various clinical endpoints, including recurrence-free survival, progression-free survival, and time to next treatment (TTNT), which are used to assess the efficacy of cancer therapies.^1^, ^2^, ^3^ These endpoints rely on the accurate documentation of recurrence/progression diagnoses in health care records. However, recurrence data is inconsistently recorded in large databases.^4^ Where the recurrence date is not recorded in a structured format, it is retrospectively inferred through manual chart review.^5^

The burden of chart review has encouraged the automated identification of recurrence diagnosis dates from structured administrative and electronic health record (EHR) data.^6^ Methods used in previous studies to identify the date of first recurrence, range from simple rules-based methods,^7^, ^8^, ^9^, ^10^, ^11^, ^12^, ^13^ to machine learning (ML) models, like decision trees,^14^, ^15^, ^16^ and logistic regression.^17^^,^^18^ Random forests,^19^ and conditional inference trees^20^ have also been used to identify patients whose cancer recurred. While the act of identifying patients who have had a recurrence alone does not enable survival analysis, this alternative output can be used for measuring population prevalence and identifying study cohorts.

In addition to the variety of automated algorithms, a range of performance statistics and thresholds have been proposed to indicate a successful algorithm, yet there has been no consensus in the literature on a single measure of success.^6^ Few studies use survival analysis as an evaluation measure,^15^^,^^16^ even though it is a major end use/research goal if their models were to be implemented.^1^, ^2^, ^3^ Finally, none of the studies identified in previous literature reviews identify the date of recurrence/progression beyond the patient’s first recurrence.^6^, ^21^

Our ambition is to create an algorithm which can detect all cancer recurrences using data routinely collected by the UK’s National Health Services and returned to the National Cancer Registration and Analysis Service (NCRAS).^22^ In this paper, we take the example of epithelial ovarian cancer (EOC) (including ovary, fallopian, and primary peritoneal cancer) and only using structured (i.e. not free text) chemotherapy data, aim to show (with common performance metrics and survival analysis) that it is possible to accurately identify multiple recurrence events using relatively simple, implementable, and interpretable ML models. Finally, we discuss the implementation and interpretability of our models with the aim that they will be implemented alongside a chart reviewer.

## Materials and methods

### Dataset description

The chemotherapy treatment histories of an initial cohort of 1996 EOC patients who received chemotherapy at Leeds Teaching Hospitals Trust (LTHT) were assessed for inclusion in this study. We selected EOC because it is a cancer which sees a majority of patients go through multiple lines of treatment and it is chemotherapy that makes up the majority of these treatments.^23^ The LTHT is a regional referral centre that supports the treatment of patients within Yorkshire and the Humber (one of the nine regions of England) and fully supports those people geographically closest to LTHT. Our first exclusion criteria on the initial cohort considered the level of curation of the programme number data type. Within the LTHT EHR, a programme number identifies the instance of progression or recurrence of each patient’s cancer that each chemotherapy regimen was being used to treat and designates the line of treatment/therapy. The chart reviewers, led by an oncology consultant, curate patients’ records for whom LTHT has a complete record of care from diagnosis. These reviewers had access to all structured and unstructured information within the patients’ EHRs when curating these programme numbers. They followed a standard operating procedure and referred any disagreement in curation to an additional oncologist for final decision. The clinical difference between a recurrence and a progression is a contested subject. We refer to the Cancer Outcomes and Services Dataset’s definition of cancer recurrence, defined as ‘the return of cancer after treatment and after a period of time during which the cancer cannot be detected’ and is only differentiated from the progression of their cancer due to the patient having ‘previously been informed that they are free of the disease or that the disease is not detectable’.^24^ Therefore, in our research, there is little interest in discriminating between the two outcomes. Consequently, throughout this paper, where a model is identifying a recurrence event, it is referring to a proxy chemotherapy treatment event following a recurrence or progression diagnosis aligning to the initial labelling system used by the chart reviewers.

**Figure 1:**
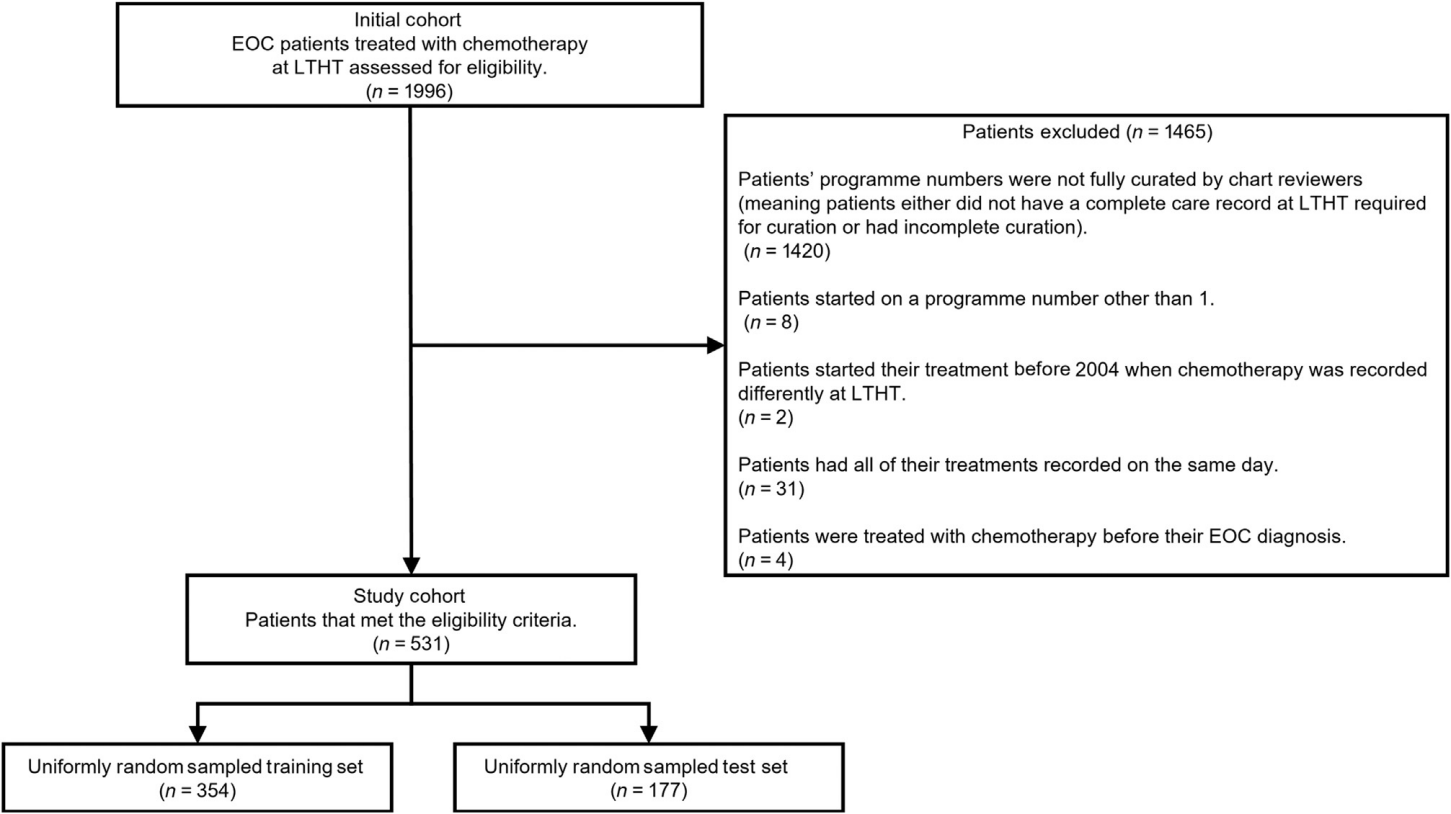
**Study population exclusion criteria.** Diagram describing the exclusion criteria used to select the study cohort from an initial cohort of EOC patients’ chemotherapy histories. EOC, epithelial ovarian cancer; LTHT, Leeds Teaching Hospitals Trust.

Following the implementation of further exclusion criteria shown in Figure 1, the initial cohort was reduced to a study cohort of 531 patients’ chemotherapy treatment histories. The study cohort comprised 6619 recorded chemotherapy treatments from 2008 to 2021. These included 127 unique drug regimens detailing the dose, schedule and supportive medication for the categorical anticancer drug therapies (including both maintenance and hormone therapy, see Supplementary material Table S1, available at https://doi.org/10.1016/j.esmorw.2024.100038) a clinician can select from a drop-down menu within the EHR. The timing of their treatments was conveyed by an associated ‘days since EOC diagnosis’ attribute. Within the EHR, the date of each treatment is recorded but this was altered to ‘days since EOC diagnosis’ to make the data suitable for research. Using the programme numbers, it is possible to quantify the two subgroups within our final cohort: patients whose cancer had not recurred/progressed (*n* = 258, ∼49%), and those whose cancer did recur/progress (*n* = 273, ∼51%).

The method of recording the drug regimen within the EHR allows for similar drug regimens to be recorded in a variety of ways [an example being ‘CARBO 1W (C)’ and ‘CARBOPLATIN 1W (C)’ both recording the regimen for weekly carboplatin], which impedes any ML method’s modelling of the relation between similar/identical drugs and recurrence events. To combat this, the 127 unique regimens were grouped into 26 clinically relevant drug regimen groups by an oncology consultant with 20 years of experience. The drug regimens and assigned drug regimen groups can be found in Supplementary Table S1, available at https://doi.org/10.1016/j.esmorw.2024.100038.

This project was approved under the IRAS Project ID: 294683 titled ‘REAL-Cancer 01: a real world evidence alliance at Leeds study to evaluate clinical characteristics, outcomes, and healthcare costs in patients with cancer’. The research was limited to the use of previously collected, non-identifiable information. Opt-out patients were not included in this study. Individual consent was not sought from the patients. This study was carried out in accordance with the Declaration of Helsinki.

### Recurrence event detection model development

Converting the programme number of each treatment into a binary label—where a change in programme number coinciding with a recurrence event would take a value of one—enables our models to use supervised learning to classify each treatment event a patient had, as either a recurrence event or not. We can then use the associated ‘days since EOC diagnosis’ of the identified recurrence event as a proxy for the date of recurrence diagnosis.

The R software was used to develop logistic regression, decision tree, conditional inference tree and random forest models to identify the treatment after a recurrence or progression diagnosis.^25^ The logistic regression models used the default binomial generalised linear model function within R.^25^ The decision tree, conditional inference tree and random forest used the rpart,^26^ party^27^ and randomForest^28^ packages, respectively.

To classify each chemotherapy event in the patients’ health records as either a recurrence event or not, seven candidate features were selected as inputs for the ML models. The first two features were the days since EOC diagnosis, and the drug regimen group of each treatment. The third feature was the integer gap in days between each treatment and their respective previous treatment. This aimed to convey that chemotherapy treatments following a recurrence/progression typically occur after a longer interval, than treatments consecutively given to treat the same instance of cancer. This feature was also intended to help the models distinguish a change in drug regimen due to toxicity and a change due to the diagnosis of a recurrence (see Supplementary material Figure S1, available at https://doi.org/10.1016/j.esmorw.2024.100038, showing the distribution of the number of days between consecutive treatments of different drug regimens).

The remaining four features were the drug regimen group and the gap between treatments for both the previous and subsequent treatment of each treatment. In the case of the first treatment, the previous gap between treatments and the previous drug regimen group was set to 0 and Not a Number (NaN), respectively, and similarly for the subsequent values of these variables for the last treatment of each patient. These four features provided the ML methods with additional context surrounding each treatment.

The study cohort was split into a uniformly randomly sampled 354 (66%)-patient training set and 177 (33%)-patient test set, while ensuring that every drug regimen group was present in both.

Since this investigation classified each treatment event, the ratio of recurrence events (∼9%) to non-recurrence events (∼91%) was highly imbalanced. The area under the receiver operating characteristic (AUROC), a common statistic recognised in clinical and computing studies, does not reflect the performance of classifiers on highly imbalanced datasets. Considering this, and the intent for our models to be implemented alongside chart reviewers to identify and suggest when a recurrence event is suspected, we value both sensitivity and positive predictive value (PPV) rather than maximising one at the expense of the other, to reduce the false identification of recurrence events requiring a chart reviewer’s attention. Therefore, the F1 score, the harmonic mean of sensitivity and PPV, was chosen as the metric to maximise when developing candidate ML models. In the absence of accepted performance thresholds in the literature, we defined the following thresholds to evaluate our models: F1 ≥ 0.95 = Excellent, F1 ≥ 0.9 = Good, F1 ≥ 0.85 = Fair, F1 ≥ = 0.8 Moderate.

We used cross-validation to develop optimised models for a random forest, conditional inference tree, decision tree and logistic regression. The models were left at their default hyperparameters for candidate variable selection (Supplementary material Table S2, available at https://doi.org/10.1016/j.esmorw.2024.100038). The models were cross-validated for a total of three iterations of 10-fold cross-validation. For each iteration of cross-validation, each of the 127 possible candidate models made from the possible combinations of the seven candidate features were trained on a nine-fold subset of the original training set and validated on the remaining fold of the original training set. We identified and retrained the best-performing candidate model of each type on the whole patient training set for testing. On the test set, we measured F1 score, overall accuracy, sensitivity, specificity, PPV, negative predictive value (NPV) and AUROC. Additionally, we calculated the percentage of identified recurrence events within ±60 days of a labelled event in positively identified recurrent patients, allowing the models comparison against papers that use a similar metric. 95% confidence intervals (CIs) were calculated with the empirical bootstrapping method over 1000 iterations of the test set to show how the models cope on a varying proportioned dataset.^29^^,^^30^

### Model application to time to next treatment

With the ‘days since EOC diagnosis’ of identified recurrence events, we can estimate TTNT survival for each instance of a patient’s cancer. We calculate TTNT as the number of days between the first chemotherapy treatment of an instance of cancer and the first chemotherapy treatment of the next instance that has been identified by the models as a recurrence event. We produced TTNT Kaplan–Meier survival curves based on the recurrence events identified by chart review and compared them to the TTNT Kaplan–Meier survival curves based on recurrence events identified by the models using the survival package in R.^31^ This provides an easily comprehensible comparison for a clinician to assess whether using the model-identified recurrence events to calculate the TTNT Kaplan–Meier survival is comparable to the TTNT survival inferred from chart review. The probabilities of a change in the line of treatment (recurrence event) at each timestep were also subjected to a log-rank test to test the null hypothesis that there was no difference between the TTNT produced using chart review-identified recurrence events and the TTNT based on a given model’s identified events.^32^

### Model application to identify patients whose cancer recurred/progressed

**Figure 2:**
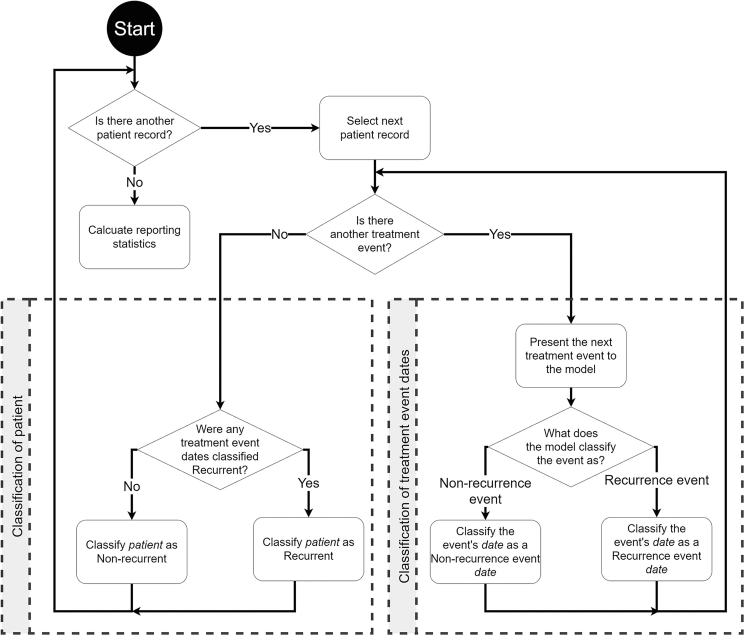
**Flow chart showing the order of classifying a patient’s treatment events using a machine learning model and the later inference of the patient’s recurrence status.** The model first classifies the treatment events of a patient enabling the use of their associated date or ‘days since EOC diagnosis’ for TTNT survival analysis. Then the existence of a model-identified recurrence event within a patient’s record is used to classify the patient as a recurrent patient. EOC, epithelial ovarian cancer; TTNT, time to next treatment.

Several previous studies’ models were optimised to identify patients as having had a recurrence, with fewer studies making the further step in estimating a date for recurrence, the step most essential for survival analysis. In contrast, our approach is optimised to identify the dates of recurrence events, but in doing so, we can infer that a patient’s cancer has recurred/progressed. We inferred the identification of patients whose cancer recurred and quantified the models’ performance on this secondary goal using F1 score, overall accuracy, sensitivity, specificity, PPV and NPV, allowing us to compare our model’s ability to identify patients whose cancer has recurred/progressed with other studies. 95% CIs were again calculated with the empirical bootstrapping method over 1000 iterations of the test set to show how the models cope on a varying proportioned dataset.^29^^,^^30^ A flowchart showing how the model first classifies the treatment events of a patient, enabling the use of their associated date or ‘days since EOC diagnosis’ for TTNT survival analysis, before any identified recurrence events are used to classify the patient as recurrent, can be seen in Figure 2.

## Results

The same candidate features maximised the F1 score for all models in all three iterations of 10-fold cross-validation: the drug regimen group used on that treatment event, the integer gap in days between the current and previous treatment, and the previous treatment’s drug regimen group. Table 1 shows the models’ F1 score, accuracy, sensitivity, specificity, PPV, NPV, AUROC and percentage of identified recurrence events within ±60 days of a labelled event in positively identified recurrent patients when detecting recurrence events in the 177-patient test set. Table 2 shows the F1 score, accuracy, sensitivity, specificity, PPV and NPV when using the models’ identified recurrence events to infer the identity of patients whose cancer recurred/progressed in the test set.

**Table 1:** Model performance for identifying when a change in the line of chemotherapy treatment occurred in the 177-patient test set

Method	F1 (95% CI)	Accuracy (95% CI)	Sensitivity (95% CI)	Specificity (95% CI)	PPV (95% CI)	NPV (95% CI)	AUROC	Percentage of identified recurrence events within ± 60 days of a labelled event (95% CI)
Random forest	0.941 (0.916-0.968)	0.990 (0.986-0.994)	0.971 (0.948-0.997)	0.992 (0.988-0.996)	0.913 (0.872-0.956)	0.997 (0.995-1.000)	0.989	94.4 (91.8-96.7)
Conditional inference tree	0.878 (0.842-0.917)	0.981 (0.976-0.987)	0.837 (0.786-0.893)	0.994 (0.990-0.998)	0.923 (0.882-0.970)	0.986 (0.981-0.991)	0.982	97.3 (95.7-99.1)
Decision tree	0.860 (0.827-0.902)	0.977 (0.971-0.984)	0.872 (0.829-0.925)	0.986 (0.981-0.991)	0.847 (0.795-0.905)	0.989 (0.985-0.994)	0.954	90.5 (86.7-93.7)
Logistic regression	0.849 (0.809-0.895)	0.978 (0.972-0.984)	0.767 (0.707-0.834)	0.996 (0.994-0.999)	0.950 (0.915-0.988)	0.980 (0.974-0.986)	0.966	97.1 (94.2-99.0)

A change in the line of chemotherapy treatment due to the diagnosis of a recurrence or progression (recurrence event) was used as a proxy for a recurrence or progression diagnosis.

AUROC, area under the receiver operating characteristic; CI, confidence interval; NPV, negative predictive value; PPV, positive predictive value.

**Table 2:** Performance of each of the final models when used to identify patients whose cancer recurred/progressed in the177-patient test set

Method	F1 (95% CI)	Accuracy (95% CI)	Sensitivity (95% CI)	Specificity (95% CI)	PPV (95% CI)	NPV (95% CI)
Random forest	0.966 (0.947-0.988)	0.966 (0.950-0.986)	0.988 (0.976-0.998)	0.946 (0.909-0.979)	0.944 (0.905-0.980)	0.989 (0.977-0.997)
Conditional inference tree	0.941 (0.911-0.980)	0.944 (0.914-0.969)	0.941 (0.901-0.979)	0.946 (0.909-0.979)	0.941 (0.900-0.977)	0.946 (0.908-0.979)
Decision tree	0.943 (0.914-0.970)	0.944 (0.914-0.969)	0.965 (0.929-0.991)	0.924 (0.882-0.964)	0.921 (0.878-0.961)	0.966 (0.932-0.991)
Logistic regression	0.952 (0.924-0.979)	0.955 (0.928-0.978)	0.929 (0.880-0.972)	0.978 (0.957-0.994)	0.975 (0.951-0.994)	0.938 (0.894-0.973)

A change in the line of chemotherapy treatment due to the diagnosis of a recurrence or progression (recurrence event) was used as a proxy for a recurrence or progression diagnosis.

CI, confidence interval; NPV, negative predictive value; PPV, positive predictive value.

The random forest model achieved the highest F1 score, both for identifying recurrence events and for identifying patients whose cancer recurred/progressed, satisfying our ‘Good’ and ‘Excellent’ thresholds, respectively. The logistic regression model achieved the lowest F1 score for detecting recurrence events, while the conditional inference tree measured the lowest for identifying patients whose cancer recurred/progressed (Table 1).

**Figure 3:**
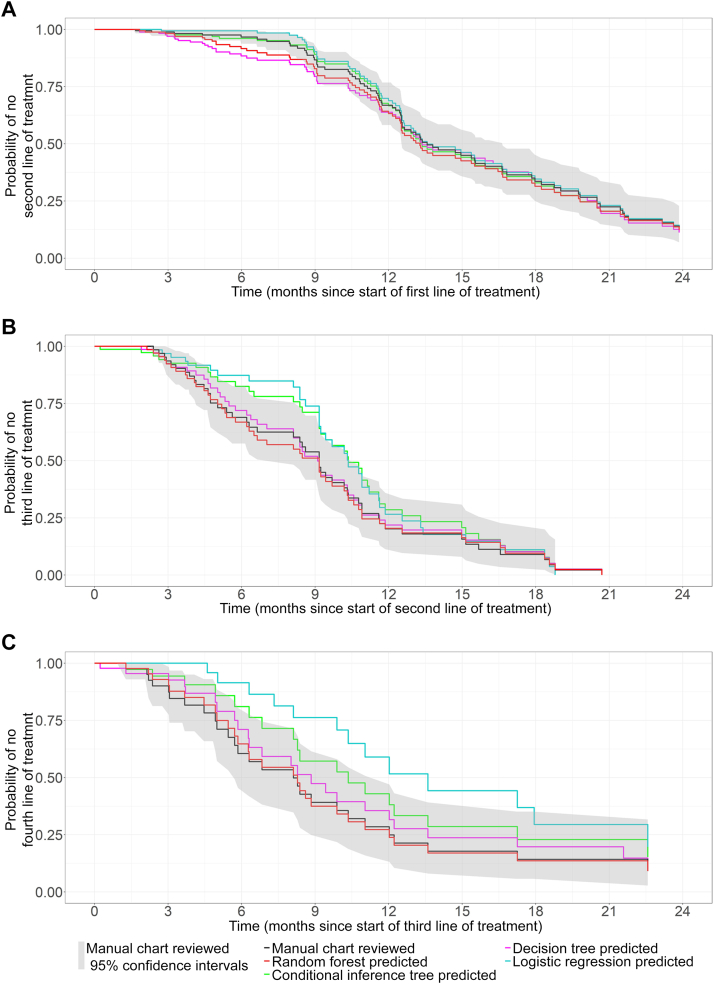
**Kaplan–Meier TTNT survival for the first three cancer recurrences.** Survival was calculated from the model-identified dates of changes in line of treatment and compared against the TTNT survival calculated from manual chart review informed dates of changes in line of treatment, for the first- (A), second- (B), and third-line (C) of treatment on the test set of 177 EOC patients. A change in the line of chemotherapy treatment due to the diagnosis of a recurrence or progression was used as a proxy for a recurrence or progression diagnosis. EOC, epithelial ovarian cancer; TTNT, time to next treatment.

Figure 3 shows TTNT Kaplan–Meier survival curves for the first, second and third changes in line of treatment (recurrence events), based on chart review and our best-performing ML models (created with the R package ggsurvfit^33^). The log-rank tests in Table 3 show that TTNTs based on model-identified dates of recurrence events were statistically significantly indistinguishable from the TTNTs based on dates identified by chart review; the only exception was the TTNT for a third recurrence event based on the best-performing logistic regression model. Both Figure 3 and Table 3 show that the conditional inference tree most closely matches with the manual chart review for the first recurrence event’s TTNT, while the random forest most closely matches with the TTNT survival for further recurrences, closely followed by the decision tree. The period in which the random forest and decision tree TTNT Kaplan–Meier survival curves deviate from the 95% CI of the manual chart review for the first recurrence event (Figure 3A) is very soon after the start of chemotherapy treatment. All models’ median TTNT survival for the first recurrence event were comparable to the chart reviewers’ estimate of 13.6 months. Only the random forest and decision tree-estimated median TTNT Kaplan–Meier survival were similar to that inferred by chart review beyond the first line of treatment (after the first recurrence event).

**Table 3:** Log-rank tests, comparing the survival probability, measured from the ML model and chart review-identified dates of change in the line of chemotherapy treatment, due to a recurrence/progression of a patient’s epithelial ovarian cancer

Model	First recurrence/progression log-rank test		Second recurrence/progression log-rank test		Third recurrence/progression log-rank test		Fourth recurrence/progression log-rank test		Fifth recurrence/progression log-rank test	
	*χ*^*2*^	*P*	*χ*^2^	*P*	*χ*^2^	*P*	*χ*^2^	*P*	*χ*^2^	*P*
Random forest	0.3	0.6	0	0.9	0	0.9	0.2	0.7	2.3	0.1
Conditional inference tree	<0.1	>0.9	1.6	0.2	1.7	0.2	0.2	0.7	0.3	0.6
Decision tree	0.4	0.6	0.1	0.8	0.4	0.5	0.3	0.6	0.4	0.5
Logistic regression	0.1	0.7	1.4	0.2	5.7	<0.1	0.4	0.5	0.5	0.5

The *χ*^2^ statistic for two curves to be considered significantly different is *χ*^2^> 3.84.

## Discussion

We evaluated the ability of four ML models to identify chemotherapy treatment dates following an EOC recurrence/progression diagnosis and by extension patients whose cancer had recurred/progressed. Here we compare our results against relevant literature.

### Identifying recurrence events

Unfortunately, we are limited when comparing our model’s performance with other studies. Firstly, all previous studies we are aware of focussed on identifying dates of the first recurrence. Secondly, these studies reported differing metrics while evaluating their models. Finally, there is currently no defined threshold for what constitutes a well-performing recurrence detection algorithm.^6^ Two notable studies, Rasmussen et al.^9^ and Chubak et al.,^14^ focussed on identifying the date of first breast cancer recurrence and reported comparable statistics and favourable results in comparison to the wider field.^6^ Rasmussen et al. verified a rules-based algorithm,^9^ while Chubak et al. proposed several decision tree models.^14^ Both Rasmussen et al. and Chubak et al. reported a percentage of first recurrences detected within 60 days of a known gold-standard date of recurrence diagnosis in positively identified recurrent patients with 76% and 82% achieved, respectively.^9^^,^^14^ These metrics are not as suitable for our data where the true date of recurrence is not known. Instead, the closest comparable metric we can measure is the percentage of model-identified recurrence event dates within 60 days of the date identified by chart review in positively identified recurrent patients. Using that metric, the lowest score achieved by any of our models was that achieved by the decision tree, with 90.5% (95% CI 86.7% to 93.7%) of model-identified dates happening within 60 days of the chart review-identified dates of recurrence in positively identified recurrent patients. However, these scores should only be interpreted alongside other metrics such as the sensitivity, as a model may identify some recurrent patients and their recurrence dates perfectly, while missing other patients’ recurrences entirely. For our models, intending for them to be used alongside a chart reviewer, we opted for F1 score as our primary metric, balancing the number of false positives needing to be reviewed while ensuring we captured a high proportion of the recurrences. However, as recognised by Jung et al., the main application of recurrence detection algorithm is enabling survival analysis.^15^ Therefore, we propose that the conformance of the models TTNT survival to that of chart review informed TTNT survival should be considered when choosing a successful model. With than in mind, the random forest and decision tree TTNT survival both conform to TTNT survival informed by chart review. The decision tree’s superior TTNT survival to the conditional inference tree is unexpected considering the latter model’s higher F1 statistic. This is an example where visualising the intended end use of the models may be more informative than reviewing classic performance statistics such as F1, sensitivity and PPV.

The TTNT survival curves show a drop in performance of the models when detecting serial (second, third, fourth and fifth line) recurrences. Reasons for this might include a diminishing association between the more unique drug regimens used in later lines of therapy and recurrence/progression outcomes. Another possible reason is the lower proportion of later-line recurrences in the dataset, causing the models to overfit to detecting first recurrences and overlook patterns specific to later recurrences. Future models developed to detect specific recurrences may combat this issue.

### Identifying patients whose cancer recurred/progressed

Our primary goal was to identify proxy dates of recurrence/progression events for review, but a derived benefit is that the models can identify patients who have had a recurrence/progression of their cancer. Both Rasmussen et al.^9^ and Chubak et al.^14^ first identified patients whose cancer recurred before proposing a proxy recurrence event date. The rules-based algorithm proposed by Rasmussen et al.^9^ recorded a sensitivity of 0.973 for identifying patients who had a recurrence of their breast cancer, whereas the high-sensitivity decision tree from Chubak et al.^14^ recorded a sensitivity of 0.96. If our results are interpreted to identify patients whose EOC had recurred/progressed, the random forest achieved a sensitivity of 0.988, higher than both studies, and our decision tree achieved a sensitivity of 0.965, putting it between the two studies. Both Rasmussen et al. and Chubak et al. used multiple modalities of treatment to identify patients whose cancer recurred.^9^^,^^14^ Our results show that high sensitivities can be achieved using only structured chemotherapy data.

### Limitations

Limitations to our models include the method of drug regimen grouping, which may not be perfectly transferable across hospitals. A more recognised method of grouping the drug regimens, like that used in the UK’s Systemic Anti-Cancer Therapy dataset curated by NCRAS would be ideal.^34^ However, we are not aware of a published static mapping table. Also, we did not investigate the sensitivity of the F1 score to different starting hyperparameters as this was not the focus of the investigation.

Additionally, as we have used real-world treatment data for our models, they are inherently vulnerable to changes in treatment practices over time. In practice, models would have to be periodically retrained to manage any changes in treatment methods.

Furthermore, ovarian cancer patients have been measured to have a 60%-80% recurrence rate.^23^ However, in our study cohort (*n* = 531), only 51% of the patients were identified by chart reviewers as having had a recurrence. We propose two reasons as to why this might be the case. The first is that in our dataset we can only detect recurrences that received further treatment. The second is that we did not require a specific length of follow-up for inclusion in our cohort, meaning that patients whose cancer had not yet recurred may have a recurrence later. We did not filter for a specific length of follow-up as our models’ intended use case is to detect recurrence events at any point during a patient’s chemotherapy treatment. We also expect that if we required a length of follow-up, we would exclude patients with aggressive cancers that recur early in a patient’s treatment. While adjusting the imbalance of patients who had a recurrence with those who did not to the expected percentage does not drastically change the imbalance respective to the number of recurrence events to non-recurrence events in our small dataset, in larger datasets this may have to be accounted for.

We used the dates of changes to the line of chemotherapy treatment as proxy dates for recurrences/progressions, but recurrences are usually diagnosed with a computed tomography scan or other investigation. Our future work will aim to identify recurrence/progression diagnosis dates more accurately by allowing the models to choose candidate variables from a broader range of modalities, including radiology results and biochemical markers, with the secondary aim of advising their inclusion into national datasets, if they enable more accurate recurrence detection and survival analysis.

### Considerations for implementation

We envision that an implemented model aiding recurrence curation would present recurrence event dates to a chart reviewer for their final decision on curation. When evaluating which method should be implemented to aid chart review, we should consider the performance, ease of implementation and interpretability of the models.

The random forest achieved the highest F1 score when identifying both recurrence events and patients whose cancer recurred/progressed, while also producing the TTNT survival curve that most closely matched those produced by chart review. However, if the level of interpretation required exceeds feature importance, the random forest becomes difficult to interpret for an end user. The decision tree closely followed the performance of the random forest, when identifying patients, and produced respectable TTNT survival curves only marginally less true to the chart review curves than that of the random forest. The final decision tree model is easier to interpret, as it only consists of eight splitting rules that can be presented to a chart reviewer. Simple tree-based models can also be quickly translated into any query-based language, facilitating implementation into whichever system staff use to review patients’ EHRs.

Therefore, the random forest and decision tree may be suitable for different environments. For implementation into existing EHR architecture to aid chart review, the decision tree may be preferable, whereas for further research into recurrence/progression where interpretation is not of great importance, the random forest will provide more accurate results.

### Conclusion

In conclusion, both the random forest and decision tree can closely match the performance of a chart reviewer when identifying proxy dates for a recurrence/progression diagnosis while only using patients’ chemotherapy treatment histories. By extension, we can estimate TTNT survival for EOC patients. We recommend the random forest model, but only if the need for model interpretation is low and the user’s system is capable of implementing it. However, the interpretability and ease of implementation of a decision tree make it an ideal choice to aid chart reviewers in correctly documenting the dates of recurrence/progression, facilitating research into cancer treatments. The methods we describe in this paper are intrinsically linked to the chemotherapy regimens used to treat EOC. However, we refrain from stating that similar methods are restricted to identifying multiple recurrences/progressions in ovarian cancers. Future work will investigate the use of additional treatment modalities to improve the detection of cancer recurrence.
