# Supplementary material for: Evaluation of machine learning methods for the retrospective detection of ovarian cancer recurrences from chemotherapy data
Source: ESMO Real World Data Digit Oncol. 2024 May 1;4:100038. doi: 10.1016/j.esmorw.2024.100038 (PMC12836628; doi:10.1016/j.esmorw.2024.100038)
Supplement: Supplemental Material [file mmc1.docx]

Supplementary Material

Table 1 shows the Drug Regimen Mapping Table defined by co-author Geoff Hall. Table 2 contains the default parameters of the models. Figure 1 shows the distribution of the number of days between consecutive treatments where the treatment has changed for non-recurrence events and recurrence events in the training set.

| Table 1: The original 127 Drug Regimens in the chart reviewed chemotherapy treatment history data set and their corresponding Drug Regimen Group assigned by a clinician. The Drug Regimens were grouped for two reasons. The first was to reduce the ambiguity between similar drug regimen types for the machine learning models. The second reason was the limit of 53 categorical variables within the R software packages that were used to develop the machine learning models. | |
| --- | --- |
| **Drug Regimen** | **Drug Regimen Group** |
| Anastrozole (C) | Hormones |
| ARIEL 3 (C) | PARPi |
| ATHENA_ RU_C1 (C) | Rucaparib/Nivolumab trial |
| ATHENA_NIV_RU (C) | Rucaparib/Nivolumab trial |
| BEV/CARBO/GEM L3 (C) | Carbo/Gem/Bev |
| BEV_ CAR3W_PAC1W (C) | Carbo/Paclitax/Bev |
| BEV15/CIS/GEM L3 (C) | Cisplatin/Gem/Bev |
| BEV7.5/PAC/CARBO (C) | Carbo/Paclitax/Bev |
| BEVA 15 maint (C) | Bevacizumab |
| BEVA 15MG/KG 6W (C) | Bevacizumab |
| BEVA 7.5 maint (C) | Bevacizumab |
| BEVA MAINT. (C) | Bevacizumab |
| BEVA/PAC/CARBO (C) | Carbo/Paclitax/Bev |
| BEVA_15MG/KG 6W (C) | Bevacizumab |
| BEVA15 CARBO GEM (C) | Carbo/Gem/Bev |
| CAR 5.0 PAC 70 (C) | Carboplatin |
| CARBO (AUC) 21D (C) | Carboplatin |
| CARBO (AUC) 28D (C) | Carboplatin |
| CARBO 1W (C) | Carboplatin |
| CARBO 21D (C) | Carboplatin |
| CARBO 28D (C) | Carboplatin |
| CARBO(AUC5)ETOP (C) | Carbo/Etoposide |
| CARBO/DOCETAXEL (C) | Carbo/Taxotere |
| CARBO/GEM L3 (C) | Carbo/Gem |
| CARBO/GEM OVARIA (C) | Carbo/Gem |
| CARBO/LIPO DOX (C) | Carboplatin/Caelyx |
| CARBO/LIPODOX A3 (C) | Carboplatin/Caelyx |
| CARBO/PAC 1W (C) | Carboplatin/Paclitaxel |
| CARBO/PAC 1W L3 (C) | Carboplatin/Paclitaxel |
| CARBO21 allergy1 (C) | Carboplatin |
| CARBO21 allergy2 (C) | Carboplatin |
| CARBO21 allergy3 (C) | Carboplatin |
| CARBO21 L1 (C) | Carboplatin |
| CARBO21 L3 (C) | Carboplatin |
| CARBO21premed L3 (C) | Carboplatin |
| CARBO28 allergy1 (C) | Carboplatin |
| CARBO28 allergy3 (C) | Carboplatin |
| CARBO28 L1 (C) | Carboplatin |
| CARBOPLATIN 1W (C) | Carboplatin |
| CAV (C) | CAV |
| CIS 25 1W L3 OP (C) | Cisplatin |
| CIS/ETOP De Wit (C) | Cisplatin |
| CIS/ETOPDeWit OP (C) | Cisplatin |
| CIS75/ETOP100 OP (C) | Cisplatin |
| CISPLAT L3 3W (C) | Cisplatin |
| CISPLAT(75)L3 OP (C) | Cisplatin |
| CISPLAT(OVA) L3 (C) | Cisplatin |
| CISPLATIN(75) OP (C) | Cisplatin |
| ETOP (De Wit) (C) | Etoposide |
| Ewings Consolidation (C) | Other |
| EWINGS Induction (C) | Other |
| ICON 8 (C) | Carbo/Paclitax/Bev |
| ICON 8 ARM A (C) | Carbo/Paclitax/Bev |
| ICON 8 ARM B (C) | Carbo/Paclitax/Bev |
| ICON 8 ARM C (C) | Carbo/Paclitax/Bev |
| ICON8B Arm B1 (C) | Carbo/Paclitax/Bev |
| ICON8B Arm B2 (C) | Carbo/Paclitax/Bev |
| ICON8B Arm B3 (C) | Carbo/Paclitax/Bev |
| ICON8B B1 PAC AL (C) | Carbo/Paclitax/Bev |
| ICON8B beva main (C) | Bevacizumab |
| LETROZOLE (C) | Hormones |
| LIPODOX/CARBO (C) | Carboplatin/Caelyx |
| LIPODOX/CARBOL3 (C) | Carboplatin/Caelyx |
| LIPOSOMAL DOXO (C) | Caelyx |
| LOGS LETROZOLE (C) | Hormones |
| LUNG MATRIX C (C) | Other |
| MEDROXPROGESTERO (C) | Hormones |
| MEGACE/TAMOXIFEN (C) | Hormones |
| MEGESTROL ACETAT (C) | Hormones |
| METRO-BIBF (6WK) (C) | BIBF |
| METRO-BIBF (C) | BIBF |
| NICCC LIP DOX (C) | Caelyx |
| NICCC PACLI (C) | Paclitaxel |
| NIRAPARIB (C) | PARPi |
| NIRAPARIB 3M (C) | PARPi |
| NIRAPARIB EAP (C) | PARPi |
| OLAPARIB (C) | PARPi |
| OLAPARIB 1ST 3M (C) | PARPi |
| OLAPARIB 3M (C) | PARPi |
| OLAPARIB 3RD (C) | PARPi |
| OLAPARIB EAP (C) | PARPi |
| OLAPARIB GYN 1ST (C) | PARPi |
| OLAPARIB GYN 2ND (C) | PARPi |
| ORZORA (C) | PARPi |
| PAC ALB GYNAE (C) | Paclitaxel albumin |
| PAC CIS(75)L3 OP (C) | Cisplatin/Paclitaxel |
| PAC L3 ALL/CARBO (C) | Carboplatin/Paclitaxel |
| PAC WK + CIS OP (C) | Cisplatin/Paclitaxel |
| PAC/CARBO 1W (C) | Carboplatin/Paclitaxel |
| PAC/CARBO 3W (C) | Carboplatin/Paclitaxel |
| PAC/CARBO 3W L3 (C) | Carboplatin/Paclitaxel |
| PAC/CARBO DD (C) | Carboplatin/Paclitaxel |
| PAC/CARBOL3 3W (C) | Carboplatin/Paclitaxel |
| PAC/CARBOL3 DD (C) | Carboplatin/Paclitaxel |
| PAC/CISL3 DD (C) | Cisplatin/Paclitaxel |
| PAC90CARBO3.3 (C) | Carboplatin/Paclitaxel |
| PAC90CARBO3.3 L3 (C) | Carboplatin/Paclitaxel |
| PAC90L3 CAR3.3 (C) | Carboplatin/Paclitaxel |
| PACL3 3W (C) | Paclitaxel |
| PACL3/CARBO 1W (C) | Carboplatin/Paclitaxel |
| PACL3/CARBO 3W (C) | Carboplatin/Paclitaxel |
| PACL3/CARBO DD (C) | Carboplatin/Paclitaxel |
| PACL3/CARBOL3 1W (C) | Carboplatin/Paclitaxel |
| PACL3/CISL3 1W (C) | Cisplatin/Paclitaxel |
| PACL3+CARBOL3 1w (C) | Carboplatin/Paclitaxel |
| PACLI CIS(75) OP (C) | Cisplatin/Paclitaxel |
| PACLI L3 1W (C) | Paclitaxel |
| PACLITAX/CARBO (C) | Carboplatin/Paclitaxel |
| PACLITAXEL 1W L3 (C) | Paclitaxel |
| PACLITAXEL 1W. (C) | Paclitaxel |
| PARP BRCA POC8 1 (C) | PARPi |
| PEM/CIS OP (C) | Other |
| PEMETREXED (C) | Other |
| PETROC ARM1 (C) | Carboplatin/Paclitaxel |
| PETROC ARM2 (C) | Carboplatin/Paclitaxel |
| PETROC Phase II study (C) | Carboplatin/Paclitaxel |
| RUCAPARIB (C) | PARPi |
| SEQ.DOUBLET(4#C/D THEN 4#P/C) (C) | Seq Doublet |
| STORM PART B C1 (C) | Other |
| STORM PARTB C2-8 (C) | Other |
| TAMOXIFEN  GYNAE (C) | Hormones |
| TAMOXIFEN GYN LD (C) | Hormones |
| TAMOXIFEN GYN MT (C) | Hormones |
| TOPOTECAN 1W (C) | Topotecan |
| TRAMETINIB GYN (C) | Trametinib |
| TRIOC (C) | Trovax vaccine |

| Table 2: The Default setting of the four models in R Software code. See respective package documentation references in main manuscript. | |
| --- | --- |
| Model | Default Settings |
| Random Forest | randomForest(x, y=NULL, xtest=NULL, ytest=NULL, ntree=500,  mtry=if (!is.null(y) && !is.factor(y))  max(floor(ncol(x)/3), 1) else floor(sqrt(ncol(x))),  weights=NULL,  replace=TRUE, classwt=NULL, cutoff, strata,  sampsize = if (replace) nrow(x) else ceiling(.632*nrow(x)),  nodesize = if (!is.null(y) && !is.factor(y)) 5 else 1,  maxnodes = NULL,  importance=FALSE, localImp=FALSE, nPerm=1,  proximity, oob.prox=proximity,  norm.votes=TRUE, do.trace=FALSE,  keep.forest=!is.null(y) && is.null(xtest), corr.bias=FALSE,  keep.inbag=FALSE, |
| Conditional Inference Tree | ctree(formula=, data=, weights=NULL, subset=NULL, control = ctree_control(), ytrafo = NULL, scores = NULL)  ctree_control=(teststat = c("quad",”max”),  testtype = c("Bonferroni", "MonteCarlo",  "Univariate", "Teststatistic"),  mincriterion = 0.95, minsplit = 20, minbucket = 7,  stump = FALSE, nresample = 9999, maxsurrogate = 0,  mtry = 0, savesplitstats = TRUE, maxdepth = 0, remove_weights = FALSE) |
| Decision Tree | Decision Tree (formula =, data=, method = 'class',weights=NULL, subset=NULL, na.action = na.rpart,model = FALSE, x = FALSE, y = TRUE, parms=NULL, control = rpart.control()))  rpart.control=minsplit = 20L, minbucket = round(minsplit/3), cp = 0.01,  maxcompete = 4L, maxsurrogate = 5L, usesurrogate = 2L, xval = 10L,surrogatestyle = 0L, maxdepth = 30L) |
| Logistic Regression | glm(formula=, data=, family=binomial, weights=NULL, subset=NULL, na.fail, start = NULL, etastart=NULL, mustart=NULL, offset=NULL, control = list(), model = TRUE, method = "glm.fit", x = FALSE, y = TRUE, singular.ok = TRUE, contrasts = NULL)  glm.control=(epsilon = 1e-8, maxit = 25, trace = FALSE) |


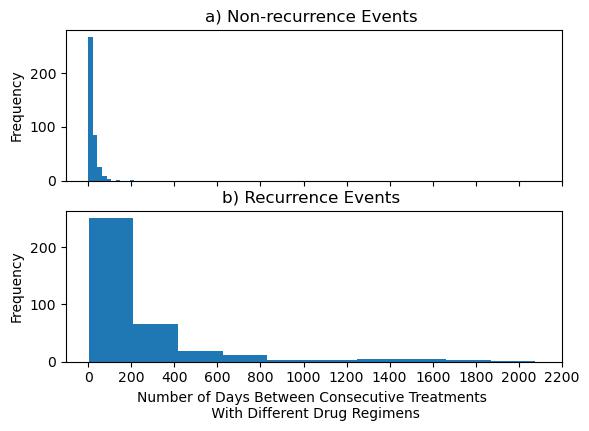


Figure 1: The distribution of the number of days between consecutive treatments where the treatment has changed for non-recurrence events (a) and recurrence events (b) in the training set. The decision to include the feature quantifying the gap between consecutive consecutive treatments was not only to capture the relation between a recurrence commonly occurring after a period of time of no treatment but also to help the models discriminate between changes in chemotherapy drug in consecutive treatments due to toxicity and changes in chemotherapy drug due to a change in line of therapy associated with a recurrence diagnosis.
